# Similarity network fusion to identify phenotypes of small-for-gestational-age fetuses

**DOI:** 10.1016/j.isci.2023.107620

**Published:** 2023-08-12

**Authors:** Jezid Miranda, Cristina Paules, Guillaume Noell, Lina Youssef, Angel Paternina-Caicedo, Francesca Crovetto, Nicolau Cañellas, María L. Garcia-Martín, Nuria Amigó, Elisenda Eixarch, Rosa Faner, Francesc Figueras, Rui V. Simões, Fàtima Crispi, Eduard Gratacós

**Affiliations:** 1 BCNatal – Barcelona Center for Maternal-Fetal and Neonatal Medicine (Hospital Clínic and Hospital Sant Joan de Deu), IDIBAPS, University of Barcelona, and Centre for Biomedical Research on Rare Diseases (CIBER-ER), Barcelona, Spain; 2 Department of Obstetrics and Gynecology, Faculty of Medicine, Universidad de Cartagena, Cartagena de Indias, Colombia; 3 Aragon Institute of Health Research (IIS Aragon), Obstetrics Department, Hospital Clínico Universitario Lozano Blesa, Zaragoza, Spain; 4 University of Barcelona, Biomedicine Department, IDIBAPS, Centre for Biomedical Research on Respiratory Diseases (CIBERES), Barcelona, Spain; 5 Faculty of Medicine, Universidad del Sinu, Cartagena de Indias, Colombia; 6 Metabolomics Platform, IISPV, DEEiA, Universidad Rovira i Virgili, Biomedical Research Centre in Diabetes and Associated Metabolic Disorders (CIBERDEM), Tarragona, Spain; 7 BIONAND, Andalusian Centre for Nanomedicine and Biotechnology, Junta de Andalucía, Universidad de Málaga, Málaga, Spain; 8 Biosfer Teslab, Reus, Spain; 9 Institute for Research & Innovation in Health (i3S), University of Porto, Porto, Portugal

**Keywords:** Public health, Pregnancy, Machine learning

## Abstract

Fetal growth restriction (FGR) affects 5–10% of pregnancies, is the largest contributor to fetal death, and can have long-term consequences for the child. Implementation of a standard clinical classification system is hampered by the multiphenotypic spectrum of small fetuses with substantial differences in perinatal risks. Machine learning and multiomics data can potentially revolutionize clinical decision-making in FGR by identifying new phenotypes. Herein, we describe a cluster analysis of FGR based on an unbiased machine-learning method. Our results *confirm* the existence of two subtypes of human FGR with distinct molecular and clinical features based on multiomic analysis. In addition, we demonstrated that clusters generated by machine learning significantly outperform single data subtype analysis and biologically support the current clinical classification in predicting adverse maternal and neonatal outcomes. Our approach can aid in the refinement of clinical classification systems for FGR supported by molecular and clinical signatures.

## Highlights

• Using an unbiased machine-learning method, we identified two clusters of human FGR
• Growth-restricted fetuses assigned to cluster A had poorer outcomes than cluster B
• Clustering of FGR using machine learning improved the prediction of preeclampsia
• The reliability of each cluster was replicated using an independent test set

## Graphical abstract

**Figure:**
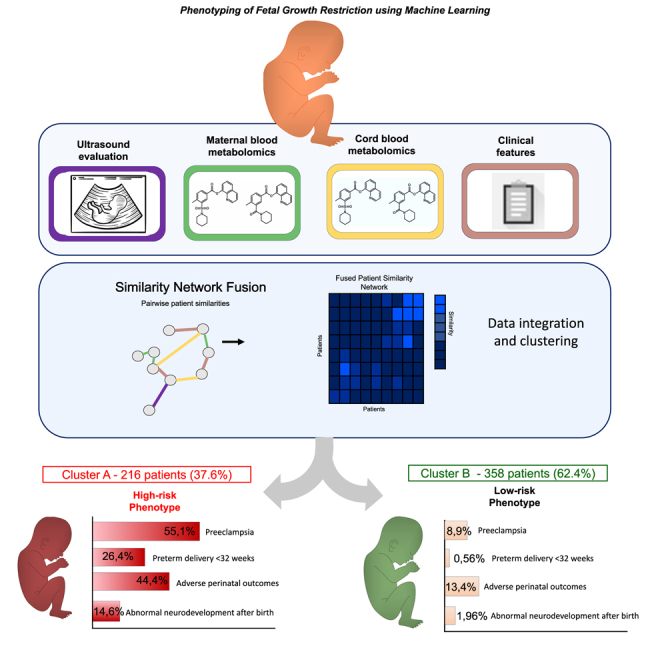

## Introduction

Fetal growth restriction (FGR), the largest contributor to fetal death,^1^ is associated with neonatal morbidity, suboptimal neurodevelopment, and chronic diseases later in life.^2^^,^^3^^,^^4^^,^^5^ Infants born with FGR have an increased risk of suboptimal neurodevelopment^6^^,^^7^^,^^8^^,^^9^^,^^10^ and are more susceptible to adult-onset chronic conditions later in life.^11^^,^^12^^,^^13^^,^^14^ FGR has no gold standard definition, and nowadays,^15^^,^^16^^,^^17^^,^^18^^,^^19^ a widely used proxy for FGR is the delivery of a small-for-gestational-age (SGA) infant (birthweight <10^th^ centile) combined with evidence of uteroplacental insufficiency or the occurrence of an adverse perinatal outcome.^15^^,^^16^^,^^17^^,^^18^^,^^19^^,^^20^^,^^21^ However, the large variability of clinical presentations and perinatal risks associated with SGA has hindered the implementation of a clinical classification for FGR since SGA includes a multiphenotypic spectrum with substantial differences in perinatal risks.^22^ A first clinical distinction has commonly been established between early onset and late onset FGR, which are associated with different severity and progression of Doppler changes.^23^ Among late onset FGR, another common distinction is established between “low-risk SGA” and “true FGR”.^23^ Indeed, a relatively large proportion of small fetuses in late pregnancy are not associated with adverse outcomes. These low-risk SGA have even been often defined and referred to as “constitutionally” small. However, this notion is not substantiated by biological evidence, and it is unclear whether low-risk SGA simply represents a clinical form with lower severity within the same condition.

The prevailing approach for discriminating between different types of FGR is based on clinical series and expert opinion-driven clinical classifications. Such approaches generally consider the severity of fetal weight reduction, growth velocity, and fetal Doppler changes to make decisions about fetal surveillance, timing, and route of delivery.^17^^,^^24^ These classification systems have demonstrated acceptable predictive ability for stillbirth and adverse perinatal outcomes.^22^^,^^23^^,^^25^ However, there is room for an inherent flaw in defining FGR based on clinical expert opinion-driven clinical criteria since these classifications do not have a biological basis to support whether they can distinguish overlapping entities or merely identify different degrees of severity within the same condition.^16^^,^^17^^,^^23^^,^^26^^,^^27^

Besides ultrasound findings, the dysregulation of angiogenesis in the placenta and maternal-fetal circulation has emerged as one of the main pathophysiological features in the development of placenta-mediated FGR.^28^ Previous studies have demonstrated that angiogenic factors in maternal circulation could potentially classify placenta-mediated fetal smallness (FGR) vs. nonpathological SGA.^29^^,^^30^ Furthermore, using metabolomics and proteomics, we and others have reported metabolic adaptations with distinct patterns in essential amino acids and abnormal lipid metabolism in mothers and growth-restricted fetuses, which are thought to persist postnatally with implications for adult disease and preventive strategies.^13^^,^^31^^,^^32^^,^^33^^,^^34^^,^^35^ These data suggest that angiogenic factors analysis and “omics” profiling could help revealing pathophysiological differences underlying the clinical phenotypes of FGR.^34^^,^^36^^,^^37^^,^^38^^,^^39^ However, parsimoniously integrating the clinical features of the condition with these different sets of biomarkers and omics data are challenging; particularly, exploiting each source of information adequately, getting a glimpse into the pathobiology, and aiding in the clinical understanding of the condition.

Several bioinformatics tools can now be harnessed to assess the heterogeneity of a condition, extending beyond classification criteria and working diagnoses.^40^ Data-driven clustering methods provide an alternative to the conventional comparison between clinically defined groups.^41^ Machine learning has the potential to revolutionize clinical decision-making and diagnosis.^42^^,^^43^^,^^44^ Recently, an unsupervised machine learning method, similarity network fusion (SNF), has been proposed to identify phenotypes integrating diverse and heterogeneous data types to distinguish patient subgroups.^45^ This machine learning method identifies homogeneous entities for different diseases, helping their characterization,^46^ with important implications for oncology,^47^^,^^48^^,^^49^^,^^50^ psychiatry,^51^ neurology,^52^^,^^53^ cardiology,^54^ and immune disorders.^40^^,^^41^^,^^55^ Given the clinical relevance of FGR and the clear need for classification systems that capture the heterogeneity of fetal smallness, we hypothesize that computational methods such as SNF may address this problem by clustering patients based on clinical and biological phenotype. This study aimed to characterize the phenotypic variability of FGR using a machine learning classification based on a multi-omics approach, combining clinical characteristics, ultrasound Doppler, metabolic/hormonal profiling, and angiogenic factors. And subsequently, to determine the predictive performance for adverse maternal and perinatal outcomes compared to the current clinical classification. Thus, we probed the existence of two different disease clusters, demonstrating that integrative analysis provides a framework for understanding human suboptimal fetal growth, with potential applications across the spectrum of human FGR.

## Results

### Baseline characteristics and perinatal outcomes of the overall cohort

In this study, 652 pregnancies were prospectively followed up based on the estimated fetal weight, of which 574 (88%) pregnancies were confirmed with a birthweight below the 10^th^ centile. Detailed maternal demographics, pregnancy characteristics, and perinatal outcomes of the included patients, as well as the training and validation cohorts, are summarized in Table 1. The median (IQR) maternal age was 33 (28–36) years. Most women were non-Hispanic white (71.7%), followed by Hispanic white (11.7%), and Asian or Pacific Islander women (10.4%). Among the patients included, 188 had preterm birth (<37 weeks, 32.8%), and 386 had term deliveries (median [IQR] gestational age at delivery of 37.5 [35.3–39.3] weeks). The rate of concomitant preeclampsia was 26.4%, and 15 cases of fetal death were documented (2.62%). As expected, no significant differences were found between the training and the validation datasets (Table 1).

**Table 1:** Maternal baseline characteristics, pregnancy characteristics, and perinatal outcomes of patients included

	All patients n = 574 (IQR or %)	Training set n = 403 (IQR or %)	Validation set n = 171 (IQR or %)	p values
**Maternal demographics**				

Maternal age (years)	33 (28–36)	33 (28–36)	33 (29–36)	0.73
Race/ethnicity				0.84
non-Hispanic white	386 (71.7)	264 (70.4)	122 (74.8)	
non-Hispanic black	11 (2)	8 (2.13)	3 (1.84)	
Hispanic white	63 (11.7)	49 (13.1)	14 (8.59)	
Asian or Pacific Islander	56 (10.4)	38 (9.4)	26 (15.2)	
Others	22 (4.1)	16 (3.9)	6 (3.68)	
Nulliparity	334 (62.2)	230 (61.7)	104 (63.4)	0.77
Pre-gestational BMI (kg/m^2^)	21.9 (20.1–25)	22.1 (20.1–25.1)	21.7 (20–24.1)	0.25

**Pregnancy characteristics**				

Antenatal clinical classification of fetal smallness				0.98
SGA	168 (29.3)	120 (29.8)	50 (29.2)	
FGR	406 (70.7)	283 (70.2)	121 (70.8)	
Preeclampsia	151 (26.4)	111 (27.6)	40 (23.4)	0.34
Gestational diabetes	35 (6.13)	27 (6.72)	8 (4.73)	0.48
Preterm birth	188 (32.8)	133 (33)	55 (32.2)	0.92

**Perinatal outcomes**				

Vaginal delivery	292 (51.2)	208 (51.9)	84 (49.7)	0.45
Emergency Cesarean section	108 (18.9)	79 (19.7)	29 (17.2)	0.56
Gestational age at delivery (weeks)	37.5 (35.3–39.3)	37.5 (35–39.4)	37.6 (36.1–39.3)	0.86
Birthweight (grams)	2280 (1774–2604)	2260 (1735–2615)	2290 (1860–2600)	0.85
Birthweight percentile	1.5 (0–5)	1 (0–5)	2 (0–4.5)	0.51
Male sex	312 (54.4)	220 (54.6)	92 (53.8)	0.94
APGAR score below seven at 5 min	22 (3.84)	14 (3.47)	8 (4.71)	0.64
Fetal death	15 (2.62)	9 (2.24)	6 (3.53)	0.93

Abbreviations: BMI = body mass index; EFW = estimated fetal weight; FGR = Fetal growth restriction; SGA = Small for gestational age.

Missing values: Maternal race (36).

### SNF identified two groups of FGR fetuses

#### Single omic networks

**Figure 1:**
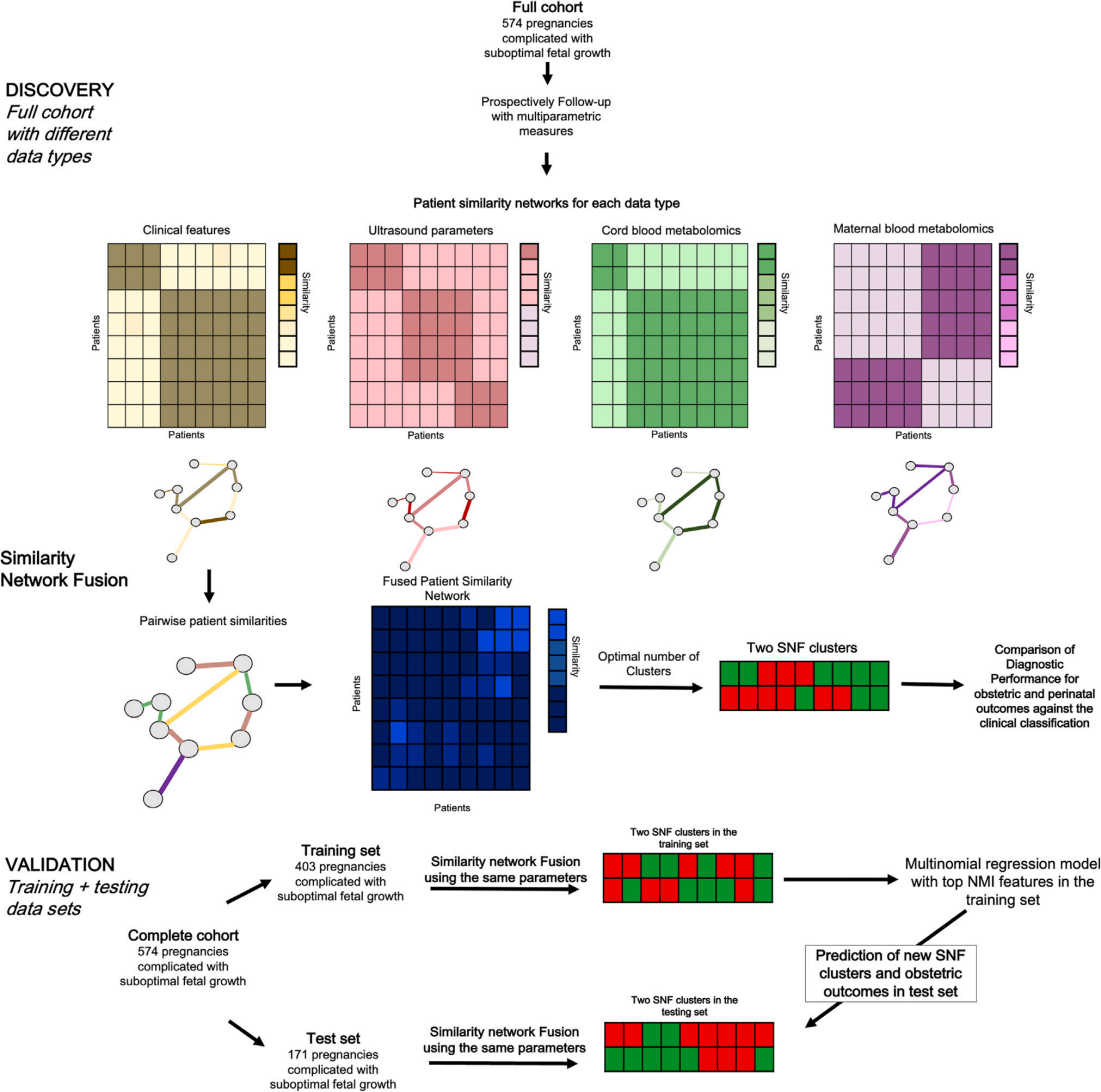
Overview of the analysis in the study The figure shows the processing phases employed in the SNF analysis. Each data type is first used as domains to construct an individual patient network to explore the inherent heterogeneity of each data layer. SNF generates patients’ similarity networks for each data type and then iteratively fuses these networks. Patients are symbolized as nodes (circles), and the similarity between their disease phenotypes is expressed as connecting edges. The number of clusters is selected based on the eigenvectors and the eigenvalues of the features included. Next, the cohort was divided into a training and testing set. Next, the SNF analysis was rerun on the training and testing sets, using the same parameters as the entire cohort, including the number of new patient groups. Finally, a classifier was constructed on the training cohort to predict group assignments to the validation cohort.

**Figure 2:**
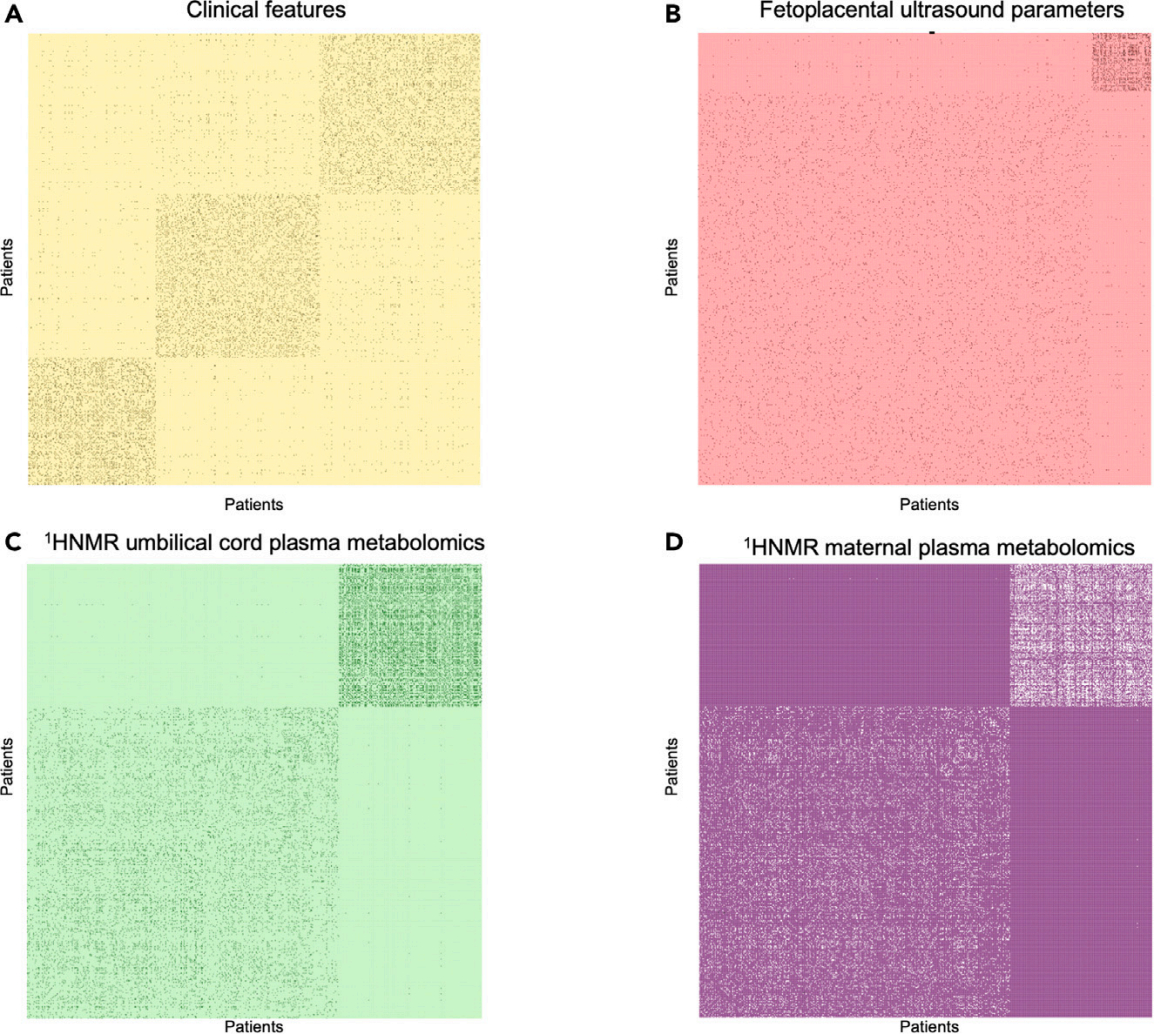
Distinct patient grouping within single omic networks (A) Heatmap of similarities between pairs of patients (both axes; same order from bottom-left) for maternal clinical features (blood pressure, angiogenic factors, and gestational age at diagnosis). The groups are visually depicted using spectral clustering that draws the groups of patients to the diagonal of the heatmap; the more substantial the similarity between patients, the closer they are grouped, and the lighter the color is shown. (B) Similarity network using maternal-fetal ultrasound (fetal biometry and maternal-fetal Doppler). (C and D) Similarity network using cord blood metabolomics, and (D). Similarity network using metabolomic fingerprinting in maternal blood.

Figure 1 outlines the analysis conducted in this study. Before applying our SNF, we performed data preprocessing, including missing-data imputation and normalization (Figure S1). Complete data were obtained for 574 pregnancies complicated with small fetuses. Each of the data types ([1] clinical features, [2] maternal and fetal ultrasound, [3] ^1^H-NMR metabolomics in umbilical cord plasma, and [4] ^1^H-NMR metabolomics in maternal plasma) was first used as domains to construct an individual patient network to explore the inherent heterogeneity of each data layer (Figure 2; Table S1). As expected, networks built using a single layer generated different patterns but supported patient similarity, suggesting at least two clusters per data type. Although the individual analysis of clinical features revealed three patient clusters (Figure 2A), integrated networks constructed with either fetoplacental ultrasound parameters, umbilical cord plasma ^1^H-NMR metabolomics, and maternal plasma ^1^H-NMR metabolomics (Figures 2B–2D) strongly support the existence of two clusters, with robust connectivity in the smaller patient cluster.

**Figure 3:**
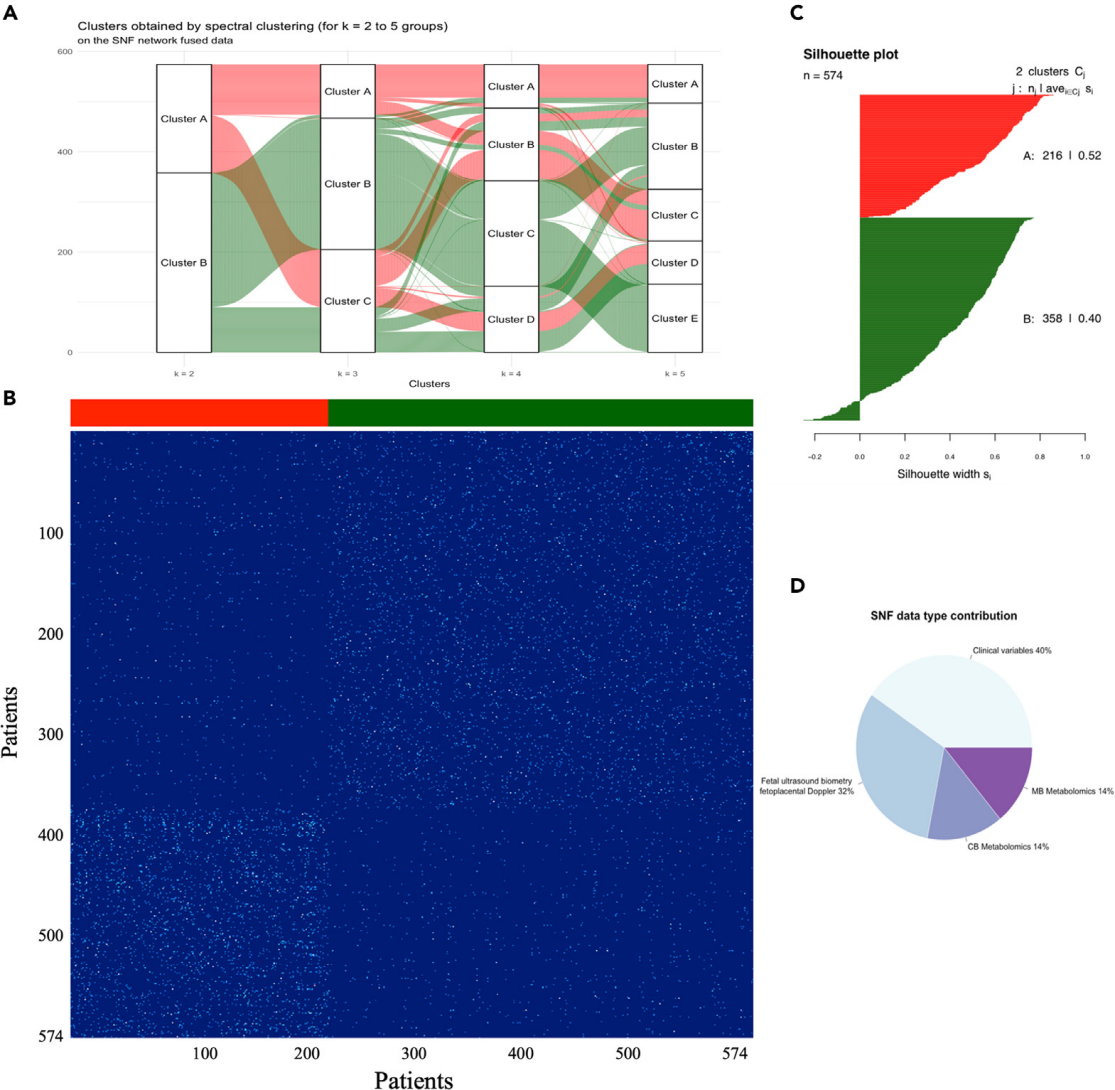
Similarity network fusion to identify new phenotypes of human fetal small for gestational age (Clusters A and B) (A) Alluvial diagram describing the process of mergers and split of clusters. Patients clustered together most similarly with two and four groups given by thick ribbons in an alluvial plot. (B) Integrated networks were combined to generate a single fused network by implementing SNF to reveal patient clusters in the entire cohort, incorporating clinical and biological data demonstrating the existence of two clusters: Cluster A included 216 patients (37.6%), whereas Cluster B included 358 patients (62.4%). (C) The silhouette score (a measure of cluster coherence) of the SNF analysis using two clusters was 0.46, demonstrating that patients were more similar within or across subtypes using two clusters. (D) SNF data type contribution. When analyzing the contribution of each component, 40% of all patient similarities (edges) were due to two data clinical features, 32% due to maternal-fetal ultrasound parameters, and the remaining SNF edges were supported by maternal and cord blood metabolomics (14% each).

Integrated networks were combined to generate a single fused network by implementing SNF to reveal patient clusters in the entire cohort, incorporating clinical and biological data (Figure 3). Two and four clusters seem to be the best selection based on the eigenvectors and the eigenvalues. Using an alluvial plot showing how patients move between new patients’ groups and considering that four and five clusters appeared like each other, we selected two as the number of optimal clusters (SNF clusters A and B) (Figure 3A). Cluster A included 216 patients (37.6%), whereas Cluster B included 358 patients (62.4%) (Figure 3B). Next, we performed the silhouette score (a measure of cluster coherence) to determine the similarity between the clusters, as shown in Figure 3C. No single data type or combination support patient similarity across SNF (Figure 3D). We found that at least two data types supported most edges: 72% of all patient similarities (edges) were due to two data types (clinical features and maternal-fetal ultrasound parameters), whereas the remaining SNF edges were supported by maternal and cord blood metabolomics (14% each). To test the influence of gestational age, an SNF analysis was performed without including this variable in the clinical features. The results demonstrated (Figure S2) the existence of the two SNF clusters independently of including gestational age at recruitment.

### Fingerprints of each cluster

**Figure 4:**
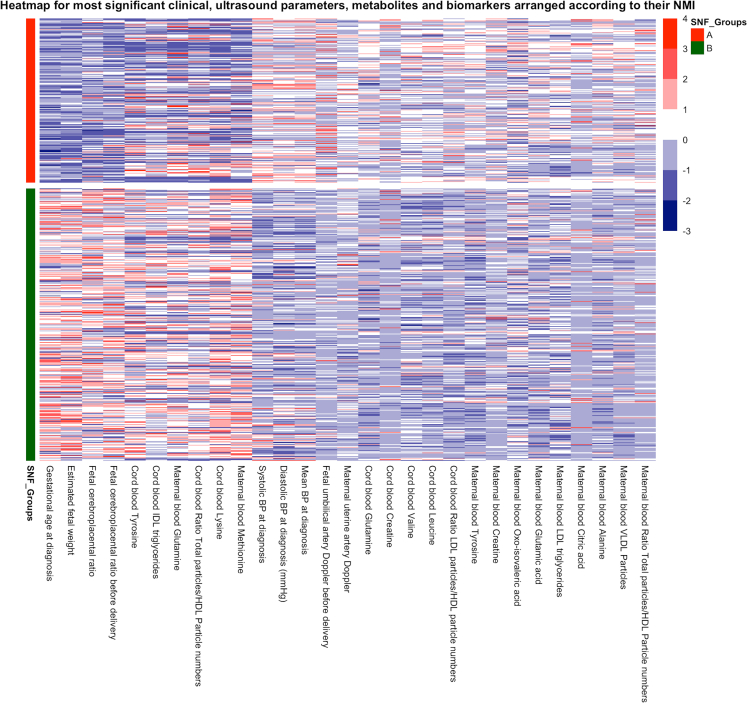
Heatmap including principal clinical, ultrasonographic, and metabolomic signatures in each phenotype The heatmap shows the 54 most variable features among the two SNF clusters (same color as Figure 3), with each column representing a feature. As shown on the scale, dark blue indicates a high concentration of a particular feature in a specific group compared to other groups.

To understand the computational basis for the segregation of the small fetuses into the clusters generated with integrated SNF clustering, we ranked the features (clinical, ultrasound, umbilical cord plasma, and maternal plasma metabolites) based on their normalized mutual information (NMI) scores, as displayed in the heatmap (Figure 4).^45^^,^^47^^,^^56^ These scores measure the relevance of each feature to the SNF fused network: those with higher NMI scores have a more significant contribution to patient similarity. Table 2 displays feature relevance according to the NMI score (Top 20). Accordingly, the most important contributor was gestational age at diagnosis, which was significantly lower in cluster A patients compared to cluster B (median (IQR): 29.9 [27.1–32.3] vs. 35.1 [32.1–37.1] weeks, p < 0.001 [Mann-Whitney U-test]). EFW, followed by umbilical artery Doppler and uterine artery Doppler, were the most important contributors to fetoplacental ultrasound parameters. Maternal blood pressure was an important contributor to the clusters, with a high NMI score, even above other biomarkers such as angiogenic markers.

**Table 2:** The top 20 features contribute to the SNF clusters according to their NMI score and their distribution in each SNF cluster

Features	NMI score	NMI rank	Cluster AN = 216(IQR or %)	Cluster BN = 358(IQR or %)	p values
Gestational age at diagnosis (weeks)	0.213	1	29.9 (27.1–32.3)	35.1 (32.1–37.1)	<0.001
Estimated fetal weight (grams)	0.180	2	1133 (±499)	1814 (±567)	<0.001
Umbilical artery PI before delivery (Z-scores)	0.160	3	0.93 (0.29–2.70)	0.13 (−0.37 - 0.53)	<0.001
Maternal systolic blood pressure (mmHg)	0.129	4	130 (119–148)	113 (107–120)	<0.001
Mean uterine artery PI at diagnosis (Z-scores)	0.126	5	2.63 (1.29–3.55)	0.23 (−0.71 - 1.25)	<0.001
Cerebroplacental ratio before delivery (Z-scores)	0.114	6	−1.90 (−3.01–−0.99)	−0.68 (−1.41 - 0.04)	<0.001
Maternal mean blood pressure (mmHg)	0.100	7	99 (90.3–111)	86.3 (81.2–92.3)	<0.001
The cerebroplacental ratio at diagnosis (Z-scores)	0.079	8	−1.45 (−2.25–−0.75)	−0.51 (−1.2 - 0.17)	<0.001
Cord blood Glutamine (mM)	0.077	9	18.2 (15.7–23.4)	15.1 (13.1–17.4)	<0.001
Cord blood Creatine (mM)	0.075	10	7.05 (5.20–9.70)	4.59 (3.36–5.61)	<0.001
Cord blood Ratio LDL/HDL particle numbers	0.072	11	21.6 (19.1–26.5)	18.2 (15.9–21.2)	<0.001
Cord blood Valine (nmol/L)	0.066	12	15.6 (13.4–18.7)	14.6 (12.9–16.6)	0.002
Maternal blood Tyrosine (mM)	0.065	13	1.40 (1.19–1.68)	1.15 (0.99–1.31)	<0.001
Maternal blood Creatine (mM)	0.065	14	4.78 (3.71–7.28)	3.52 (2.70–4.46)	<0.001
Maternal blood 2-oxoisovaleric acid (mM)	0.063	15	0.75 (0.48–1.09)	0.77 (0.56–0.96)	0.78
Maternal diastolic blood pressure (mmHg)	0.063	16	84 (76–93.8)	73 (67–80)	<0.001
Cord blood Triglycerides IDL (mg/dL)	0.057	17	6.01 (3.99–8.9)	4.45 (3.15–5.77)	<0.001
Maternal blood Glutamic acid (mM)	0.056	18	1.92 (1.44–2.64)	1.62 (1.18–2.08)	<0.001
Maternal blood Triglycerides LDL (mg/dL)	0.054	19	32.2 (24.9–43.6)	33.8 (27.1–42.1)	0.48
Cord blood Tyrosine (mM)	0.054	20	2.03 (1.83–2.35)	1.81 (1.54–2.04)	<0.001

Abbreviations: HDL = High-density lipoprotein; IQR = interquartile range; LDL = Low-density lipoprotein; NMI = normalized mutual information; PI = Pulsatility Index; SNF = Similarity Network Fusion.

Regarding ^1^HNMR-based metabolomics, the most important contributors were cord blood glutamine and creatine, which were significantly higher in cluster A than in cluster B fetuses (p < 0.001, Table 2, [Mann-Whitney U-test]). In contrast, tyrosine, creatine, and 2-oxo-isovaleric acid were the most significant contributors to maternal plasma (p < 0.001). Then, the umbilical cord plasma of cluster A neonates had significantly higher concentrations of lipoproteins LDL, and VLDL triglycerides, LDL-cholesterol, as well as medium-sized VLDL and large-size LDL particles compared to cluster B fetuses. Moreover, small metabolites in cluster A neonates had significantly higher umbilical cord plasma concentrations of ethanol, methanol, threonine, creatine, glutamine, valine, leucine, and isoleucine tyrosine than cluster B.

### Demographic and pregnancy characteristics of the two SNF clusters

Women in cluster B, who deliver earlier in gestation (Table 2), were younger and presented significantly higher lower maternal pre-gestational BMI than cluster A (21.6 [19.8–23.9] vs. 22.7 [20.7–27.1] kg/m^2^, p < 0.001 [Mann-Whitney U-test]). Cluster A debuted earlier in gestation with a median (IQR) of 29.2 (27.1–32.3) weeks at diagnosis (Table 3).

**Table 3:** Maternal demographics, pregnancy characteristics, and perinatal outcomes of patients according to each cluster generated by SNF

	Cluster A N = 216 (IQR or %)	Cluster B N = 358 (IQR or %)	p values
**Maternal demographics**			

Maternal age (years)	34 (30–37)	32 (27–35)	<0.001
Race/ethnicity			<0.001
non-Hispanic white	134 (63.5)	252 (77.1)	
non-Hispanic black	8 (3.79)	3 (0.92)	
Hispanic white	39 (18.5)	24 (7.34)	
Asian or Pacific Islander Chinese	18 (5.7)	38 (11.6)	
Others	12 (5.69)	10 (3.06)	
Smoking	49 (23.6)	88 (26.7)	0.47
Nulliparity	137 (65.6)	197 (60.1)	0.24
Pre-gestational BMI (kg/m^2^)	22.7 (20.7–27.1)	21.6 (19.8–23.9)	<0.001
Assisted reproductive technology	21 (9.7)	17 (4.75)	
Gestational age at diagnosis (weeks)	29.9 (27.1–32.3)	35.1 (32.1–37.1)	<0.001

**Pregnancy characteristics**			

Preeclampsia	119 (55.1)	32 (8.96)	<0.001
Gestational diabetes	15 (6.98)	20 (5.62)	0.63
Abnormal Fetoplacental Doppler	167 (79.5)	116 (33.3)	<0.001
Prenatally classified as SGA	28 (13)	140 (39.1)	<0.001
Prenatally classified as FGR	188 (87)	218 (60.9)	<0.001

**Perinatal outcomes**			

Gestational age at delivery (weeks)	34.3 (32.0–37.1)	38.1 (37.3–39.9)	<0.001
Preterm delivery (<37 weeks)	149 (69)	39 (10.9)	<0.001
Early preterm delivery (<32 weeks)	57 (26.4)	2 (0.56)	<0.001
Cesarean section	138 (64.2)	104 (29.3)	<0.001
Emergency cesarean section	68 (31.6)	40 (11.3)	<0.001
Birthweight (grams)	1600 (1125–2150)	2450 (2190–2709)	<0.001
Birthweight centile	0 (0–2)	3 (1–6)	<0.001
Female sex	98 (45.5)	164 (45.8)	0.99
APGAR score <7 at 5 min	21 (9.77)	1 (0.28)	<0.001
Low cord blood pH at birth (<7.2)	23 (11.9)	11 (3.26)	<0.001
Cord blood BNP (mg/dL)	43.5 (15.6–130)	14.6 (8.14–25.8)	<0.001
Cord blood Erythropoietin (mg/dL)	43.4 (18.7–136)	26.2 (15.4–54.5)	0.003
Abnormal neonatal Brazelton Score	9 (14.8)	2 (1.96)	0.003
Perinatal death	15 (6.94)	0	<0.001

Data are median (interquartile range) or N (%).

Abbreviations: BMI = body mass index; BNP = B-type natriuretic peptide; NBAS = Neonatal Behavioral Assessment Scale.

Missing values: Maternal race (36).

### Perinatal outcomes of the two SNF clusters

**Figure 5:**
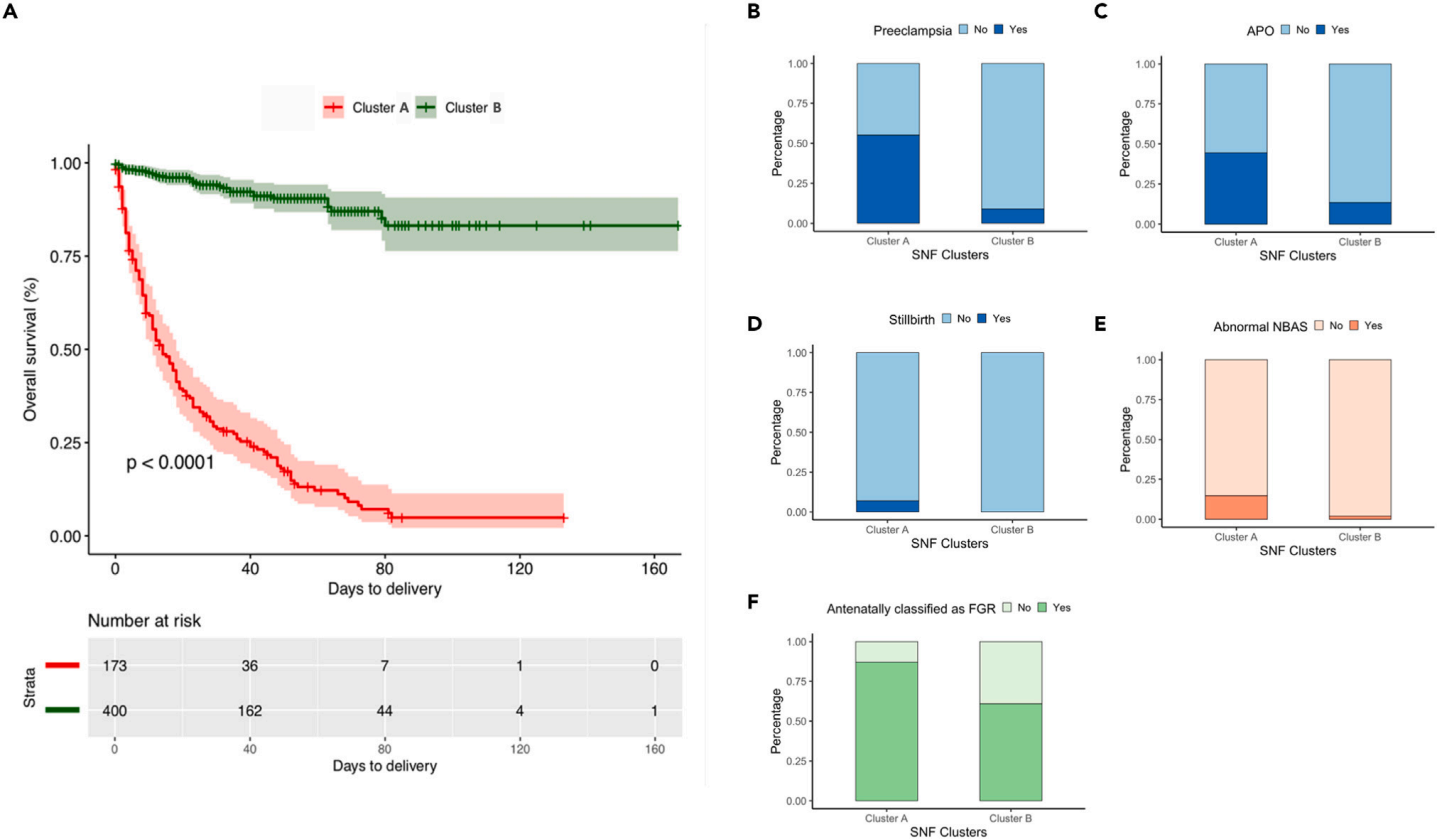
Perinatal outcomes of each phenotype of fetal small for gestational age according to the labels generated from the similarity network fusion **(**A) Kaplan-Meier curves of overall survival for each SNF subtype. The p-value was determined with a log rank test. Survival probability (interval-to-delivery) according to each cluster. (B–F) Bars represent the rate of preeclampsia (B), the occurrence of an adverse perinatal outcome (C), cases of fetal death (D), abnormal NBAS (E), and cases labeled as FGR using the clinical classification (F).

SNF subtypes exhibited more heterogeneous perinatal outcomes (Table 3; Figure 5). The largest cluster (cluster B, n = 358) included patients with better perinatal outcomes than the smaller cluster (cluster A, n = 216). Survival analysis revealed that cluster A pregnancies had poorer outcomes, with a shorter interval between diagnosis and delivery than cluster B (hazards ratio [HR] = 10.5 95% CI: (7.32–14.9), Cox log rank test p = 0.001; Figures 5A and S3). Consequently, the rate of preterm delivery before 32 weeks was significantly higher in cluster A than in cluster B (26.4% vs. 0.56%, p < 0.001, Table 3). In addition, the birthweight percentile was significantly lower in neonates of cluster A compared to cluster B (0 [0–2] vs. 3 [1–6], p < 0.001 [Fisher's exact test]). Patients in cluster A were significantly more affected by preeclampsia (55.1% versus 8.9%, p < 0.001, [Fisher's exact test]) (Figure 5B), greater prevalence of adverse perinatal outcomes (44.4% vs. 13.4%, p < 0.001 [Fisher's exact test]) (Figure 5C), and importantly, all cases of perinatal death (6.94%, p < 0.001 [Fisher's exact test]) (Figure 5D). Regarding cord blood serum, neonates in cluster A had significantly higher erythropoietin and B-type natriuretic peptide (BNP) concentrations than cluster B (Table 3, all p values < 0.05 [Mann-Whitney U-test]). BNP has been used clinically as a myocardial stress marker in patients with cardiomyopathy, ischemic heart disease, and other critical conditions. In the umbilical cord, high BNP concentrations predict neonatal cardiac dysfunction soon after birth.^57^ With regards to newborn’s responses to the extrauterine environment, the prevalence of an abnormal Brazelton neonatal behavioral assessment scale was significantly higher in cluster A than in cluster B neonates (14.7% vs. 1.96%, p < 0.001 [Fisher's exact test]) (Figure 5E). Using the combination of EFW and Doppler results, we categorized the patients using the current clinical classification in the following groups: 1) constitutional SGA (168 [29.3%]) and 2) high-risk FGR (406 [70.7%]). Figure 5F describes the crossing between the two classifications; in cluster A, 218 (87%) were classified antenatally as FGR, while 218 (60.9%) were classified as FGR in cluster B.

### Cluster validation

**Figure 6:**
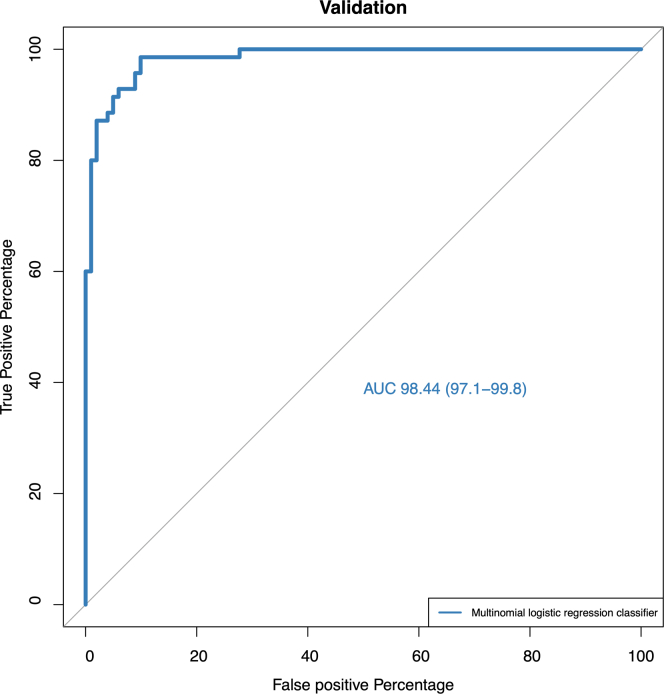
Receiver operating characteristic curve of the model’s predictive performance, constructed with the training dataset to predict new patient groups (Generated by SNF) in the testing set

Having established new groups in the entire cohort during the discovery phase, we subdivided our data into training (n = 403 patients) and testing sets (n = 171 patients). First, SNF was performed using the same parameters in the training and testing dataset and matched the new patient groups between the two cohorts. As with the discovery cohort, no group had more or fewer patients than the others (accuracy = 85.7% [SD: 0.03]). Next, a multinomial logistic regression classifier was constructed, using the variables with the highest top NMI in the training dataset to predict SNF groups in the testing dataset (Table S2). The resulting model, trained in the training cohort, recovered SNF groups well in the validation cohort (Figure 6). The model has areas under the ROC curve (AUROC) of 0.98 (95% CI 0.97–0.99).

### Diagnostic performance of SNF clustering compared with the clinical classification

**Figure 7:**
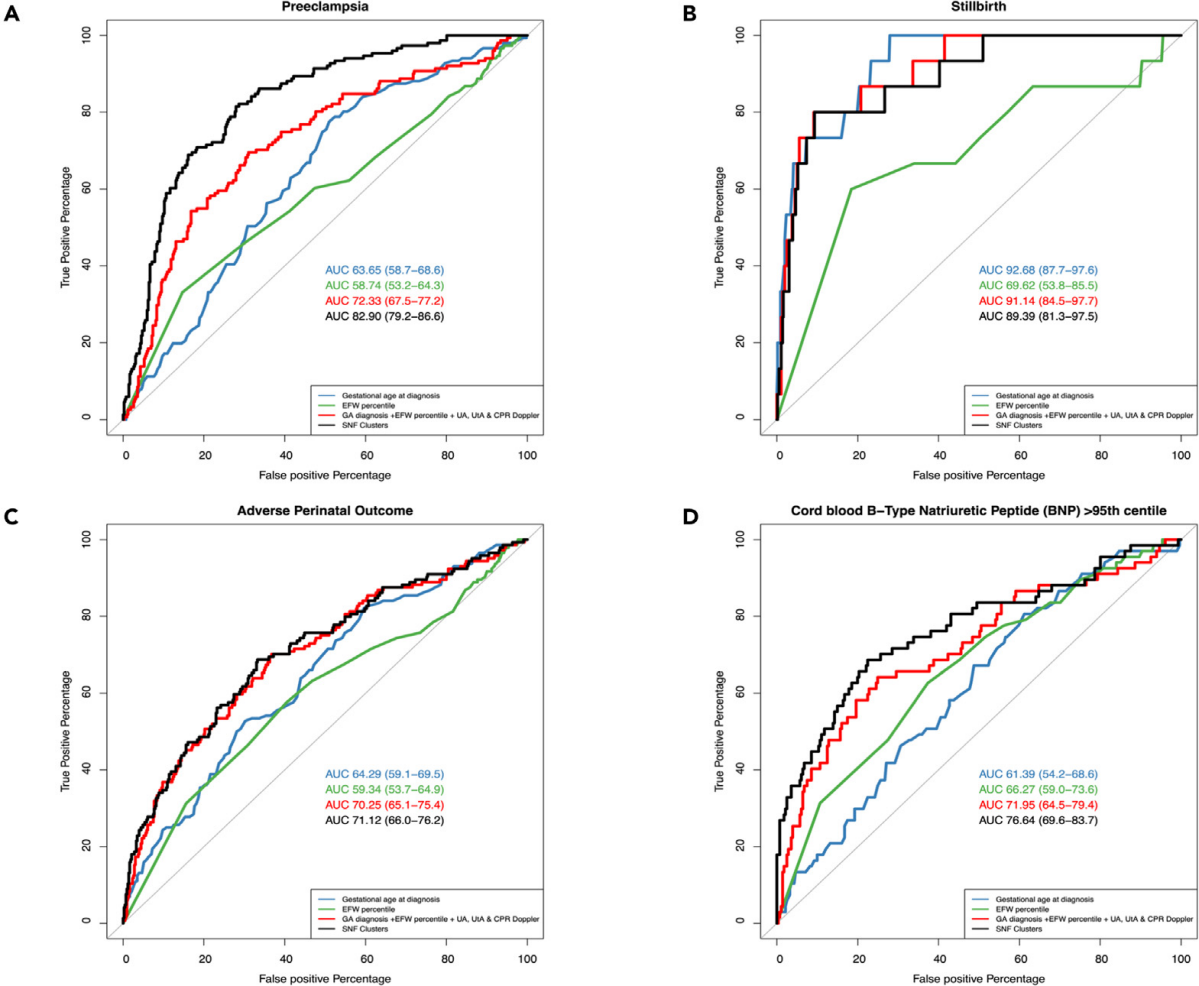
Receiver operating characteristic curve (ROC) of the gestational age at diagnosis, EFW centile, clinical classification, and the SNF clusters for each obstetric and perinatal outcome (A) Shows the ROC curves describing the predictive performance of SNF clusters (black) to identify the development of preeclampsia, as compared with gestational age at delivery (blue), EFW percentile (green), and a logistic regression model combining each parameter used in the clinical classification (red). SNF clusters consistently improved the prediction of preeclampsia over the clinical classification. (B) Shows the prediction of stillbirth, demonstrating a similar performance between GA at delivery (blue), SNF clusters (black), and the combination of clinical parameters (red). (C) Displays the ROC curves describing the predictive performance of adverse perinatal outcomes, demonstrating that SNF clusters (black) improved the prediction over gestational age at delivery (blue) and EFW percentile (green). (D) Displays the prediction of a cord blood B-type natriuretic peptide (BNP) above the 95^th^ centile. SNF clusters (black) demonstrated a better predictive performance over the other clinical parameters.

Having established the differences between the clinical classification and the newly generated SNF clusters, we next assessed their diagnostic efficacy for specific obstetric outcomes. SNF clustering had the highest diagnostic odds ratios (DOR) (Table 4). Importantly, the positive likelihood ratio of the SNF clustering for stillbirth was 2.78 (95% CI: 2.82–4.16), and women classified as mild SGA using the clinical classification had one case of fetal death (false negative). Cluster B (Lowest risk) ruled out stillbirth with a 100% negative predictive value [95% CI: 99–100]. With regards to obstetric and perinatal outcomes (preeclampsia, stillbirth, APO, and abnormal BNP concentrations in CB), SNF cluster membership probability consistently improved the prediction of preeclampsia over the clinical classification and had a similar performance for perinatal outcomes based on the area under the receiver operating characteristic (ROC) curve (AUC), which was estimated using 2,000-fold bootstrapping to account for overfitting (Figure 7; Table S3). Moreover, SNF clusters improved the prediction of each outcome over gestational age and EFW for predicting preeclampsia, APO, and abnormal BNP in cord blood (Figure 7; Table S3). Importantly, The AUC of the SNF clustering membership probability has a better performance than a logistic regression model combining each parameter used in the clinical classification (GA at recruitment, EFW, and fetoplacental Doppler) for preeclampsia 0.83 (95% CI: 0.79–0.87) vs. 0.72 (0.67–0.77), p *< 0.001* [DeLong test] Table S3). In terms of perinatal outcomes, the AUCs of the SNF clustering membership probability and a logistic regression model combining each parameter used in the clinical classification for the prediction of perinatal death, APO, and abnormal cord blood BNP were 0.89 (95% CI: 0.81–0.97), 0.71 (95% CI: 0.66–0.76), and 0.76 (95% CI: 0.69–0.84) vs. 0.91 (95% CI: 0.85–0.98), 0.70 (95% CI: 0.65–0.75), and 0.72 (95% CI: 0.64–0.79), respectively (Table S3).

**Table 4:** Diagnostic effectiveness of clinical classification and SNF clustering of small fetuses for specific obstetric and perinatal outcomes

Classifications	Outcomes	TP/FP	TN/FN	Positive LR (95% CI)	Negative LR (95% CI)	Sensitivity (95% CI)	Specificity (95% CI)	PPV (95% CI)	NPV (95% CI)	DOR (95% CI)
Clinical classification (SGA & FGR)	Preeclampsia	142/263	159/9	1.51 (1.39–1.64)	0.16 (0.08–0.3)	94 (89–97)	38 (33–42)	35 (30–40)	95 (90–98)	9.54 (4.73–19.2)
	Stillbirth	14/392	167/1	1.33 (1.15–1.54)	0.22 (0.03–1.49)	93 (68–100)	30 (26–34)	3 (2–6)	99 (97–100)	5.96 (7.78–45.7)
	APO	127/279	151/17	1.36 (1.24–1.49)	0.34 (0.21–0.53)	88 (82–93)	35 (31–40)	31 (27–36)	90 (84–94)	4.04 (2.34–6.96)
	Abnormal BNP in cord blood	62/174	107/5	1.49 (1.33–1.68)	0.20 (0.08–0.46)	93 (83–98)	38 (32–44)	26 (21–32)	96 (90–99)	7.62 (2.97–19.6)
SNF clustering (Clusters A & B)	Preeclampsia	119/97	325/32	3.43 (2.83–4.16)	0.28 (0.20–0.38)	79 (71–85)	77 (73–81)	55 (48–62)	91 (88–94)	12.46 (7.93–19.6)
	Stillbirth	15/201	358/0	2.78 (2.49–3.11)	0 (0 – NA)	100 (78–100)	64 (60–68)	7 (4–11)	100 (99–100)	NA
	APO	96/120	310/48	2.39 (1.97–2.89)	0.46 (0.36–0.59)	67 (58–74)	72 (68–76)	44 (38–51)	87 (83–90)	5.17 (3.44–7.75)
	Abnormal BNP in cord blood	43/46	235/24	3.92 (2.85–5.39)	0.43 (0.31–0.59)	64 (52–76)	84 (79–88)	48 (38–59)	91 (87–94)	9.15 (5.1–16.5)

The total number of women in this analysis was 574, including 151 cases of preeclampsia, 15 cases of stillbirth, and 144 cases of adverse perinatal outcomes (APO). Cord blood (CB) B-type natriuretic peptide (BNP) was measured in 348 neonates, and in 67 cases, the CB BNP concentration was above the 95^th^ centile. The proportions of sensitivity, specificity, positive predictive value (PPV), and negative predictive value (NPV) are given in percentages (%). DOR, diagnostic odds ratio; FN, False-negative; FP, false positive; LR, likelihood ratio; TN, true negative; TP, true positive.

## Discussion

### Main findings of the study

This study aimed to develop a classification system based on machine learning (SNF), providing a more comprehensive and biological basis for identifying clinical subtypes within the diagnosis of fetal smallness. Using an unbiased machine-learning method, we identified two clusters with distinct molecular and clinical features, emerging from a combination of clinical, ultrasound, angiogenic, and metabolomic analysis. Cluster A represented a smaller group of patients with an earlier onset in gestation, with a higher rate of maternal and fetal complications and a lipogenic metabolic profile consistent with a catabolic state. Cluster B represented approximately 62% of the population, comprising patients diagnosed later in gestation, with a lower rate of maternal preeclampsia and perinatal outcomes associated with milder metabolic adaptations in mothers and infants. The most relevant factors to both clusters were gestational age at diagnosis, EFW, umbilical artery Doppler, maternal blood pressure, and selected metabolites from the umbilical cord and maternal plasma. The reliability and robustness of the SNF clusters were further verified with (1) a training set of patients via bootstrap sampling and then with (2) an independent test set.

### Characteristics of FGR phenotypes using SNF

Our machine-learning approach provides a complementary perspective for understanding FGR prognosis, with remarkable similarities and differences compared with the clinical standard.^26^^,^^58^ Particularly, the SNF classification obtained showed important resemblances, albeit with some differences, with the clinical distinction between early and late onset FGR. However, SNF did not distinguish any further cluster, and therefore it did not reproduce the commonly used clinical distinction between low-risk SGA and true FGR. Instead, SNF supported a classification where gestational age is an important but not the only one. Cluster A features are consistent with a subset of fetuses with pronouncedly impaired maternal uteroplacental perfusion, presenting 79.5% rate of abnormal fetoplacental Doppler at diagnosis and a 6.94% perinatal death rate.^3^ We believe this cluster includes mainly cases identified today as early onset FGR, in which the transfer of nutrients and oxygen to the developing fetus is severely affected, leading to higher perinatal morbidity and mortality rates.^3^^,^^59^ Indeed, early onset FGR is characterized by maternal vascular malperfusion of the placenta, abnormal transformation of the spiral arteries, and defective extravillous trophoblast invasion causing massive lesions of the placental structure,^60^^,^^61^ as well as multifocal infarction – a hallmark of placental insufficiency.^62^^,^^63^^,^^64^

Concerning the relationship between the clusters and the other common clinical classification of “low-risk SGA and true FGR”, 63% of the fetuses in Cluster B would have been clinically termed FGR, and 37% SGA. While late onset FGR is also considered a placental disease, the placental lesions leading to impaired transference of nutrients and oxygen are milder,^65^ and the fetal hemodynamic compromise is less severe and different compared to early onset FGR.^23^ However, within late onset small fetuses, the clinical distinction between late onset FGR and SGA is relevant due to differences in clinical evolution and prognosis.^66^^,^^67^ In essence, SGA presents rates of adverse pregnancy outcomes similar to normal fetuses, while FGR is associated with more severe outcomes.^68^ Interestingly, Cluster B failed to pick up these differences in clinical evolution, suggesting that Cluster B fetuses, irrespective of their low-risk SGA or true FGR clinical classification, shared a common biological basis. This aligned with a large body of evidence demonstrating that most late onset small fetuses, regardless of whether they are classified as SGA or FGR, have remarkable pathophysiological similarities, including similar changes in fetal metabolism,^37^ brain remodeling and microstructure,^69^^,^^70^ cardiovascular programming,^71^^,^^72^ and accelerated placental aging.^73^ Thus, when analyzed from an unbiased biological perspective, SGA and FGR appear to be different forms of severity within the same condition. Therefore, the present results support previous evidence suggesting low-risk SGA are not “constitutionally small” fetuses despite their association with normal obstetrical outcomes.^67^^,^^74^

### Integrative SNF approach

While omics data are comprehensive, they can generate noisy data if used independently, often as heterogeneous as the condition itself, or provide an inconsistent grouping of individuals. Here, the resulting network has two clusters as two layers, but the clinical and ultrasound are different in number and type of clusters. We performed clustering by integrating untargeted umbilical cord plasma and maternal plasma ^1^HNMR metabolomics with clinical and ultrasonographic data. Regarding ^1^HNMR metabolomics, the most important contributors were umbilical cord plasma glutamine and creatine, which were significantly higher in cluster A than in cluster B fetuses. Glutamine is the most abundant free amino acid in the body. It is essential for pH homeostasis, nucleotide synthesis, and protein anabolism.^75^^,^^76^ Our data is consistent with previous studies demonstrating that expression of glutamine’s transporter proteins (LAT1, LAT2, SNAT5, and EAAT1) was increased in the placentas of growth restricted versus AGA infants.^77^ On the other hand, several metabolites of energy production and cell proliferation pathways are included.^78^ Concerning ^1^HNMR maternal plasma metabolomics, tyrosine, creatine, and 2-oxo-isovaleric acid were the most important contributors, reflecting renal dysfunction since these mothers were more affected by preeclampsia.

Our unsupervised analysis is aligned with previous studies suggesting that maternal adaptations through gestation, such as blood pressure or the hemodynamic profile, could be more accurate in predicting FGR than angiogenic markers.^34^ However, most studies combine biological data by concatenating normalized measurements from various biological domains, such as mRNA expression and DNA methylation. Previous studies have exploited the use of machine learning models in predicting FGR have been reported.^79^^,^^80^^,^^81^^,^^82^^,^^83^^,^^84^^,^^85^ While some have focused exclusively on maternal characteristics,^82^^,^^85^ others have employed ultrasound parameters,^79^^,^^80^^,^^81^^,^^84^ or biomarkers on the maternal blood^83^ in the first or second trimester. While analyzing ultrasound parameters such as the umbilical artery PI^79^^,^^84^ (similar performance to ours) and second-trimester fetal nuchal fold have emerged as strong predictors of FGR,^80^^,^^81^ a more integrated approach to the prediction of FGR using machine learning might be combining clinical characteristics and biochemical markers. For instance, a recent study reported a model with acceptable performance for predicting SGA neonates in the first trimester.^83^ Yet, reported machine learning methods have only improved prediction accuracy by approximately 10–20% compared to standard clinical diagnosis.^81^^,^^85^

Machine learning approaches have also been reported to improve the current understanding of FGR physiopathology.^86^^,^^87^^,^^88^^,^^89^^,^^90^ A recent study assessed fetal heart rate signals in 102 late FGR fetuses, suggesting their relevance as a screening tool in FGR.^90^ Another study harnessed support vector machine learning algorithms combining ^1^HNMR and DI-LC-MS/MS biochemical profiling of cord blood serum with clinical and demographic information to identify potential metabolite biomarkers of growth-restricted fetuses.^88^ While no diagnostic improvement was reported, the authors also supported the role of abnormal lipid metabolism, potentially secondary to chronic hypoxia, as a key player in the physiopathology of the condition.^88^ Concerning the placenta and its underlying molecular mechanisms, artificial neural networks, and evolutionary algorithms have identified IL-6, IGF-II, and IGFBP-2 in placenta lysates as the most important determinants of fetal growth.^91^ Another recent case-control study, 36 placenta samples harnessed machine learning with unsupervised clustering for transcriptomics and methylomic data analysis, suggesting the existence of multiple sub-phenotypes in the FGR.^89^ Moreover, gene annotation clustering revealed an association between cell signaling and proliferation and lipid metabolism with insulin resistance.^89^ Multi-omics analysis of an *in vivo* model, based on mRNA and miRNA expression in fetal liver tissues, further demonstrated significant transcriptomic and metabolomic changes in the energy and lipid metabolism during FGR.^90^ Finally, machine learning algorithms have also been proposed for assessing FGR effects during childhood, based on 24-h ECG and blood pressure monitoring and other socioeconomic attributes.^92^

The novelty of our results derives from integrating multi-modality measurements to identify similarities between patients and avoid unnecessary noise. This is paramount since fetal adaptations to a hostile intrauterine environment are underlined by metabolic changes and hormone production, and tissue sensitivity to the latter affects organ development, altogether leading to modifications in transcriptional factors and metabolic and homeostatic mechanisms.^11^ The strength of SNF, compared to other clustering techniques, relies on each data point (particular patient) contributing to the network analysis, regardless of missing values.^45^ Our analysis resulted in coherent clusters according to the silhouette score, achieving significance in survival analysis across the spectrum of the two clusters. In addition, we compared the similarity between uni- and multimodal clustering assignments using NMI scores, demonstrating high overlap for ultrasound and clinical features and the relevance of parameter selection based on NMI scores for clinical staging.^40^

### Clinical relevance

The diagnosis of FGR remains a matter of debate in modern obstetrics, given its heterogeneity and complexity.^16^^,^^26^^,^^93^ At present, several classifications can be used to define clinical subtypes in FGR.^23^^,^^26^ The clustering method evaluated here integrated objective information and supported the clinical distinction between early and late onset FGR. Specifically, clusters generated by SNF significantly outperformed single data subtype analysis and biologically support the current clinical classification in predicting adverse maternal and neonatal outcomes. Theoretically, an SNF cluster classification of FGR could be extended to clinical practice. While we acknowledge the challenge of including such a large panel of metabolomic markers and angiogenic factors, their individual contributions (NMI scores) to the classification could provide selection criteria for future clinical application, i.e., assigning higher weights to variables more determinant for outcome prediction could improve the identification of small fetuses at higher risk of complications. Aside from a clinical application, clustering methods could contribute to a better characterization and compatibility between specific clinical subsets in research studies.

### Limitations of the study

The strengths of our study rely on a well-characterized, prospective cohort based on comprehensive phenotypic data for studying pregnancies with fetuses affected by suboptimal growth. Our unsupervised method can identify similarities among individuals and deliver automated insights that might help with clinical tasks such as health monitoring, opportune delivery, and even postnatal follow-up. In addition, an unsupervised machine-learning method can analyze data much faster than any human. This includes data useful for antenatal care, such as clinical data, imaging, and angiogenic factors, and information that can help predict post-natal cardiac dysfunction and suboptimal neurodevelopment after birth aiding selective metabolites in maternal and cord blood.

We also acknowledge some limitations. First, our population was mainly white women from the Mediterranean, which might not represent global ethnical diversity. Second, the platforms used to construct the SNF, such as cord blood metabolomics, would hardly be used in the clinical setting as prediction variables due to difficulties accessing the cord blood sample. However, including fetal blood offers information on fetal status and phenotype, thus providing a unique understanding of fetal responses to placental failure. As cord blood biomarkers would be difficult to be incorporated into clinical practice, future studies could investigate prognostic factors for cluster prediction (using the identified biomarkers earlier in pregnancy and/or in other samples) with clinical utility. Third, we lacked placenta histopathology data, which could have provided additional information about the clinical phenotype. Thus, future studies aimed at ascertaining the underlying mechanisms of placental dysfunction and FGR should consider including placental RNA-seq, placental methylation, and proteomics. Finally, clinical classification is performed using both SGA and FGR. However, a limitation of our comparison is that gestational age should be considered a continuum. Therefore, we carried out a supplementary analysis in which the performance of the clinical classification was enhanced. Moreover, we included characteristics and biomarkers that were relevant at the time but might have missed additional biomarkers potentially relevant for cluster analysis, such as specific proteins,^94^ cell-free RNA,^95^^,^^96^ or placenta pathology.^97^

To conclude, unveiling and elucidating the underlying pathways of multiphenotypic diseases is required for developing new diagnostic tests and therapeutic interventions. Using an unsupervised and unbiased machine learning method based on integrative molecular and clinical analysis, our results *confirm* the existence of two subtypes of human FGR. These findings provide a biological basis for the classifications used in clinical practice. Further work should include long-term studies of each cluster, follow-up on their clinical outcome, and external validation.

## STAR★Methods

### Key resources table

**Table:** 

REAGENT or RESOURCE	SOURCE	IDENTIFIER
**Biological samples**		

Metabolomics in maternal blood	Biobanks of the Hospital Clinic-IDIBAPS and Hospital Sant Joan de Deu, Barcelona, Spain	www.ebi.ac.uk/metabolights/MTBLS8256
Metabolomics in cord blood	Biobanks of the Hospital Clinic-IDIBAPS and Hospital Sant Joan de Deu, Barcelona, Spain	www.ebi.ac.uk/metabolights/MTBLS8256

**Critical commercial assays**		

Placental growth factor (PlGF)	R&D Systems Europe Ltd, Abingdon, UK	Cat# PDPG00; RRID: D12S1900
Soluble fms like tyrosine-1 (sFlt1)	R&D Systems Europe Ltd, Abingdon, UK	Cat# PDVR100C; RRID: AB_2827807
B-type natriuretic peptide (BNP)	Siemens Healthineers	Siemens ADVIA® Centaur® BNP assay; RRID: AB_10772132

**Deposited data**		

Raw and analyzed data	This manuscript	ISCIENCE-D-23-04003
De-identified human clinical information, ultrasound and Doppler parameters	This manuscript	https://doi.org/10.5281/zenodo.8176372

**Software and algorithms**		

SNFtool	R studio	https://github.com/maxconway/SNFtool
^1^H-NMR fingerprinting analysis	Biosfer Teslab	Liposcale test

### Resource availability

#### Lead contact

Further information and request for clinical information and resources should be directed to and will be fulfilled by the lead contact, Fatima Crispi (fcrispi@clinic.cat).

#### Materials availability

Maternal and cord blood metabolomics data have been deposited in MetaboLights and are accessible through MetaboLights (accession number MTBLS8256) and are publicly available. Accession numbers are listed in the key resources table. This study did not generate new unique reagents.

### Experimental model and study participant details

In this study, 652 pregnancies with antenatal suspicion of fetal smallness were prospectively followed up at the Department of Maternal-Fetal Medicine of BCNatal (Hospitals Clínic and Sant Joan de Déu, Barcelona) based on the estimated fetal weight, of which 574 (88%) pregnancies were confirmed with a birthweight below the 10^th^ centile and were included. The median (IQR) maternal age was 33 (28–36) years. Most women were non-Hispanic white (71.7%), followed by Hispanic white (11.7%), and Asian or Pacific Islander women (10.4%). Approval was obtained from our Institutional Research and Ethics Committee (review board 2014/7154). Written informed consent was obtained from each participant. According to our institutional protocol, cases with signs of severity (EFW centile below the 3^rd^ centile, abnormal uterine artery Doppler, or abnormal cerebroplacental ratio) were delivered electively between 37 and 38 weeks of gestation, while small fetuses without signs of severity were allowed to deliver up to 40 weeks of gestation spontaneously.^22^^,^^23^^,^^98^

### Method details

#### Clinical features

The prespecified clinical features selected as input variables for the SNF analysis included gestational age at diagnosis, maternal blood pressure, and angiogenic profile (Table S1). Gestational age was calculated based on fetal crown-rump length measured by ultrasound at 11–13 weeks. The birthweight percentile was calculated using local standards for fetal sex and gestational age.^99^ Maternal demographics, pregnancy characteristics, and perinatal outcomes were prospectively recorded in all patients by interviews and revision of electronic medical records. Maternal characteristics were collected when the estimated fetal weight was below the 10^th^ centile, and a maternal-fetal ultrasound was performed. Exclusion criteria included clinical signs of intra-amniotic infection, fetal chromosomal abnormalities, or major structural abnormalities. According to standard procedure, the maternal blood pressure was measured automatically with a calibrated OMRON M6 Confort device (OMRON Corporation, Kyoto, Japan). Blood pressure was measured in one arm (right or left) without distinction, while women were seated after a 5-min rest. Mean arterial pressure (MAP) was calculated as diastolic blood pressure+(systolic blood pressure – diastolic blood pressure)/3. Maternal plasmatic concentrations of placental growth factor (PlGF) and soluble fms like tyrosine-1 (sFlt1) were measured at delivery using an ELISA kit (R&D Systems Europe Ltd, Abingdon, UK). The PlGF kit had a 7 pg/mL to 1000 pg/mL measuring range and a run control coefficient of variation (CV%) of 3.2%. The laboratory personnel were blinded to the patient’s clinical results or outcomes.

#### Fetoplacental ultrasound parameters

Transabdominal ultrasound, including maternal-fetal Doppler evaluation, was performed in all cases at diagnosis and before delivery, using 6-4-MHz probes (Siemens Sonoline Antares, Siemens Medical Systems, Malvern, PA, USA) and a Voluson 730 Expert Machine (GE Medical Systems, Zipf, Austria). EFW was calculated using the Hadlock formula,^100^ and transformed to EFW centile and EFW z-scores according to fetal gender and gestational age using local standards.^99^ Longitudinal growth assessment was performed by calculating EFW z-velocity as EFW z-score at diagnosis - EFW z-score at second-trimester evaluation (18–24 weeks)/interval between scans (days).^101^ Maternal-fetal Doppler included: umbilical artery-pulsatility index (PI), mean uterine artery PI, ductus venosus PI, and middle cerebral artery PI. In addition, the cerebroplacental ratio was calculated as middle cerebral artery PI/umbilical artery PI. The Z-scores for each Doppler vessel were then calculated according to the expected PI for each gestational age in normal pregnancies after log-or square transformation of the PI.^102^^,^^103^ Doppler evaluation at the diagnosis, and the last ultrasound before delivery were considered for the analysis. According to our institutional protocol, cases with signs of severity (EFW centile below the 3^rd^ centile, abnormal uterine artery Doppler, or abnormal cerebroplacental ratio) were delivered electively between 37 and 38 weeks of gestation, while small fetuses without signs of severity were allowed to deliver up to 40 weeks of gestation spontaneously.^22^^,^^23^^,^^98^

#### ^1^H-NMR metabolomics in umbilical cord plasma

^1^H-NMR metabolomics analysis was performed on cord blood samples obtained from the clamped umbilical vein immediately after delivery and maternal plasma (peripheral blood) collected at delivery under non-fasting conditions, at least eight hours after the last meal. Blood sampling and processing were performed within one hour, and plasma samples were frozen at −80°C. Plasma samples were thawed overnight and prepared for nuclear magnetic resonance (NMR) analyses according to the Bruker-specific metabolomics protocol.^104^ Aliquots from each sample (300 μL) were 1:1 mixed with sodium phosphate buffer for immediate analysis. High-resolution ^1^H-NMR spectroscopy data were acquired on a Bruker 600 MHz Spectrometer (Bruker Biospin, Rheinstetten, Germany) equipped with an Avance III console and a TCI CryoProbe Prodigy: 1D Nuclear Overhauser Effect Spectroscopy (NOESY), Carr-Purcell-Meiboom-Gill (CPMG), and 2D j-resolved spectroscopy (JRES), all with pre-saturation of the residual water peak for detection of small molecules such as amino acids and sugars; and 1D Diffusion, to detect larger molecules such as lipoproteins, glycoproteins, and choline compounds.^105^^,^^106^ ^1^H-NMR fingerprinting analysis, including Liposcale for lipoprotein characterization and phosphatidylcholines compounds and glycoprotein peak deconvolution, were all performed as reported before.^37^^,^^107^^,^^108^^,^^109^^,^^110^

#### B-type natriuretic peptide in umbilical cord

After the cord was clamped, umbilical cord ethylenediaminetetra-acetic acid-treated blood was obtained. Plasma was separated by centrifugation at 1500 g for 10 minutes at 4°C, and samples were immediately stored at −80°C until assayed. B-type natriuretic peptide (BNP) concentrations were measured using Siemens ADVIA® Centaur® BNP assay.^111^ This assay is indicated for the measurement of plasma BNP as an aid in diagnosing heart failure.

#### Clinical outcomes

Four investigators, J.M., C.P., L.Y., and F.C., personally reviewed the medical records. The maternal characteristics and the ultrasound data were directly recorded when the mother signed the informed consent as a part of this protocol. The additional data extracted from the medical records were mainly maternal and perinatal outcomes: delivery data and the occurrence of maternal or perinatal complications. The hospital medical records used an SAP NetWeaver system that guarantees the traceability and robustness of the introduced data. Preeclampsia was defined as high blood pressure (systolic blood pressure ≥ 140 mmHg and/or diastolic blood pressure ≥ 90 mmHg on two occasions, at least four hours apart), developed after 20 weeks of gestation, with proteinuria (≥ 300 mg/24 h) or protein/creatinine ratio ≥ 0.3.^112^^,^^113^ Adverse perinatal outcomes (APO) were defined as stillbirth, emergency operative delivery (vaginal operative delivery or Cesarean section) owing to non-reassuring fetal status, low Apgar score, or the presence of neonatal metabolic acidosis. Non-reassuring fetal status was defined as an abnormal fetal heart rate tracing and abnormal fetal scalp blood pH during intrapartum monitoring. Apgar scores < 7 at 5 min were considered low. Neonatal metabolic acidosis was defined as UA pH <7.15 and base excess in the newborn >12 mEq/L.^114^ Newborns underwent the Neonatal Behavioral Assessment Scale (NBAS) at 40 ± 1-week corrected age by observers accredited by The Brazelton Institute (Harvard Medical School, Boston, MA). The examination consisted of six behavioral areas rated on a 1–9 scale, where nine is the best performance for some areas and represented by the central score of five for other areas.^115^

### Quantification and statistical analysis

#### Data preprocessing

Before applying our SNF, we performed data preprocessing, including missing data imputation and normalization. We investigated the extent of incomplete data on all variables included in the analysis model and the plausibility of the type of missing values. Clinical data such as gestational age and estimated fetal weight was complete for all data, while blood pressure was missing in 66/574 values (11.4%), ultrasound data was missing in 24/574 values (4.1%), and metabolomic data was missing in 181/574 values (31.3%). We used multiple imputations to manage missing data by creating plausible imputed data sets based on the observed data. Then, we used the mice R package (version 2.3.0) to fit the model of appropriately combining the resulting dataset,^116^ and generating 50 imputed data sets. Figure S1 shows the percentage of imputation of each variable. Specifically, the most significant percentage of missing values were found in the metabolomic profile, EFW velocity, and ductus venosus (35% missing). The number of imputations was chosen based on the fraction of incomplete cases, as advocated by Bodner.^117^^,^^118^

#### Similarity network fusion analysis

Similarity network fusion (SNF) was applied to the clinical and biological data from 574 pregnancies complicated with suboptimal fetal growth using the SNF R package (v.2.3.0).^45^ Briefly, four domains were used to characterize the patient cohort: 1) clinical features, 2) maternal-fetal ultrasound, 3) cord blood metabolomics, and 4) maternal blood metabolomics (all SNF input features listed in Table S1). We selected the variables using a combination of approaches. First, clinical criteria such as gestational age and ultrasound are standard evaluations used in daily practice and therefore are essential for diagnosing and managing suboptimal fetal growth.^23^ For the biomarkers, we based our panel on a literature review and angiogenic markers' association with adverse outcomes in patients with preeclampsia and placental insufficiency. Concerning the other biomarkers used simultaneously in maternal and cord blood, we supported our approach on previous experiences suggesting metabolomics^37^ and BNP^119^ have important implications in the pathophysiology of the disease. The SNF cluster analysis pipeline is summarized in Figure 1. First, a patient similarity matrix was calculated for each data type, measuring how similar patients were to each other by Euclidean distances. In the second step, these patient similarity matrixes were fused. The network fusion step uses a non-linear method that iteratively updates every network, making them more resemblant after each iteration, with nodes as patients and weighted edges as pairwise patient similarities. Next, spectral clustering was performed on the fused patient similarity matrix using the built-in ‘SNFtool’ function. Finally, the affinity matrices constructed with each data type were aggregated to generate a fused patient network and a fused patient similarity matrix.

The optimal number of clusters was estimated using Eigen-gaps and rotation cost methods. The eigen-gap algorithm,^48^ and the rotation-cost algorithm^120^ were used based on the network’s connectivity to decide the optimal number of clusters in the fused network.^121^ We calculated the latter based on the eigenvectors (distance between consecutive eigenvalues of the resulted network) and sorted the eigenvalues in ascending order, determining two as the best number of clusters, followed by four clusters. Spectral clustering was then applied to delineate groups based on participant similarity matrices. A silhouette plot quantified the similarity between patients within a given group compared to patients in all other groups. Finally, we obtained the normalized mutual information (NMI) scores of the features (Table S4). The resulting NMI represents the ratio of correctly classified subjects according to the results of the SNF, where 0 equals all subjects misclassified, and 1 equals all subjects correctly classified.

#### Cluster validation

The analysis was repeated for the validation step, which included a random subsampling of the entire cohort (n = 574) into a training set (n = 403, 70.2%) and testing set (n = 171, 29.8%). To evaluate the reproducibility of the groups generated in the complete cohort, the entire SNF analysis was rerun in the training and testing set using the same parameters in the entire cohort. Next, a classifier using the top 20 NMI features derived from the SNF analysis in the training dataset was constructed using multinomial logistic regression to predict new SNF groups generated in the test set (Table S2). Logistic regression was used to model nominal outcome variables, in which the log odds of the outcomes are modeled as a linear combination of the predictor variables. We created a classifier to identify cluster A in the testing dataset using a logistic regression model constructed using the variables with the highest top NMI in the training dataset (Table S2).

#### Comparison with the current clinical classification

By combining EFW and Doppler, small fetuses were subdivided according to our current clinical classification: those with a birthweight <3^rd^ centile and/or abnormal uterine artery Doppler and/or abnormal umbilical artery Doppler and/or abnormal cerebroplacental ratio were termed FGR.^23^^,^^98^ On the other hand, those fetuses who subsequently had a birthweight between the 3^rd^ and the 9^th^ percentile and had normal fetoplacental Doppler were considered SGA cases.^23^^,^^98^ Next, diagnostic performance (sensitivity; specificity; positive and negative likelihood ratio; and the diagnostic odds ratio) was estimated and used to compare the accuracy of the two classifications (SNF-based cluster vs. groups in the clinical classification [SGA and FGR]) to identify neonates at risk of preeclampsia, stillbirth, APO, or abnormal cord blood BNP. Finally, we compared the likelihood and diagnostic odds ratios by bootstrapping 2000 replicates with replacement. The performance for predicting preeclampsia, stillbirth, APO, and abnormal BNP of gestational age and EFW as sole predictors, a regression model combining gestational age, EFW, and Doppler parameters, and the SNF cluster classification was determined by the receiver–operating characteristics (ROC) curve analysis. The resulting areas under the ROC curves (AUCs) were compared using the DeLong method, and p < 0.05 was statistically significant.

#### Statistical analysis

All statistical analyses were performed with R, version 3.6.1 (R Foundation for Statistical Computing). Categorical data are presented as n (%) and continuous data as mean (±SD) or median [interquartile range (IQR)] according to their distribution. Proportions were compared with Fisher's exact or chi-square test to assess the categorical variables. Distributions of continuous variables were examined for normality using the Kolmogorov-Smirnov test. When continuous variables were normal, the one-way ANOVA and unpaired t-tests were used to compare differences. Otherwise, the Kruskal-Wallis one-way analysis of variance and Mann-Whitney U-test were used. The Kaplan–Meier method was used to determine cumulative probabilities of being delivered from SGA diagnosis through follow-up, according to each SNF-generated cluster, with between-group comparisons of cumulative event rates calculated by means of the log rank test. Multivariate Cox proportional-hazards regression analyses were used to evaluate the time-to-delivery in each cluster from enrollment through follow-up. The Cox model was adjusted for relevant clinical covariates using best-subset regression modeling (including gestational age at recruitment). To test the hazard proportionality, the function coxph of the surv.function of the R package was applied to test the proportionality of the predictors included in the model (gestational age at diagnosis and SNF clustering) by creating interactions with log(time) (see Figure S3). All statistical tests were two-sided, and a p value of less than 0.05 was considered to indicate statistical significance.

## Data Availability

De-identified human clinical information, ultrasound, and Doppler parameters are publicly available (Zenodo data: https://doi.org/10.5281/zenodo.8176372). Accession numbers are listed in the key resources table. This paper does not report an original code. Any additional information required to reanalyze the data reported in this paper is available from the lead contact upon request.

## Supplemental information

Document S1. Figures S1–S3 and Tables S1–S3

Table S4. Features contributing to the similarity network fusion clusters according to their normalized mutual information scores and their distribution in each cluster, related to STAR Methods
