# Supplementary material for: Similarity network fusion to identify phenotypes of small-for-gestational-age fetuses
Source: iScience. 2023 Aug 12;26(9):107620. doi: 10.1016/j.isci.2023.107620 (PMC10485038; doi:10.1016/j.isci.2023.107620)
Supplement: Document S1. Figures S1–S3 and Tables S1–S3 [file mmc1.pdf]

iScience, Volume *26*

Supplemental information

Similarity network fusion to identify phenotypes of small-for-gestational-age fetuses

Jezid Miranda, Cristina Paules, Guillaume Noell, Lina Youssef, Angel Paternina-Caicedo, Francesca Crovetto, Nicolau Cañellas, María L. Garcia-Martín, Nuria Amigó, Elisenda Eixarch, Rosa Faner, Francesc Figueras, Rui V. Simões, Fàtima Crispi, and Eduard Gratacós

SUPPLEMENTARY INFORMATION

Figure S1. Visualizing the missing data.

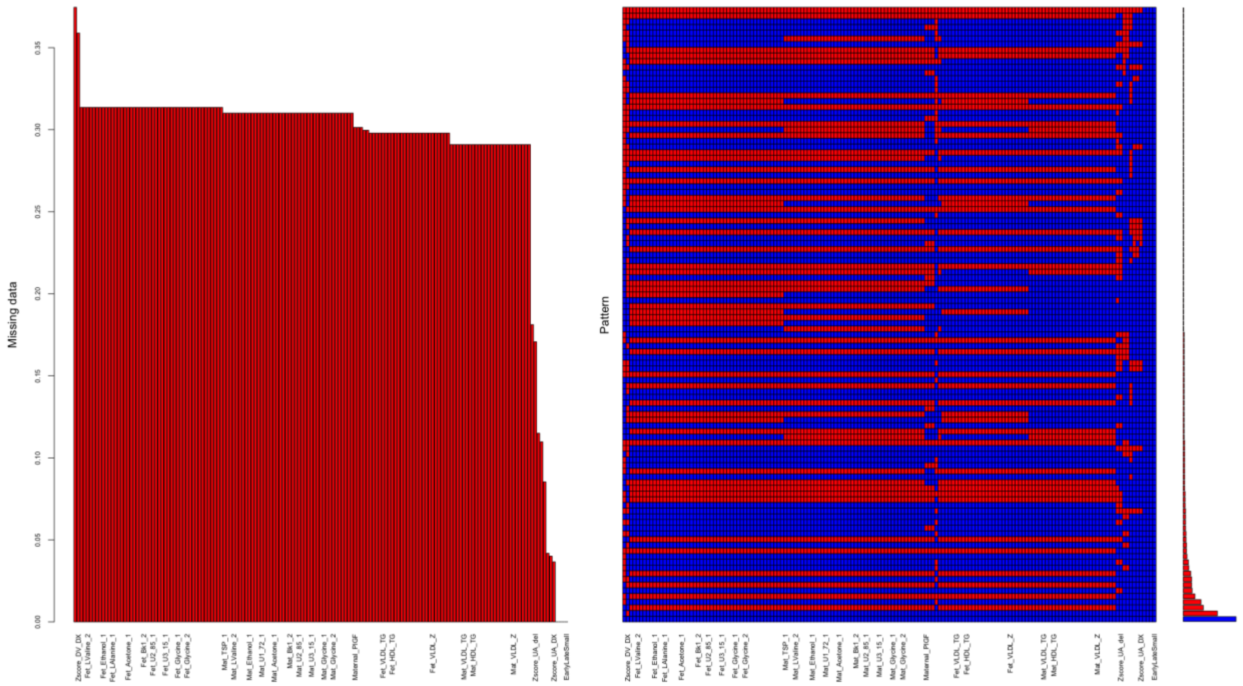

Figure S1: Missing plot describing the percentage of the imputation of each variable employed to construct SNF matrices, related to STAR methods.

Figure S2. Similarity network fusion to identify new phenotypes of human fetal small for gestational age without considering the gestational age at recruitment.

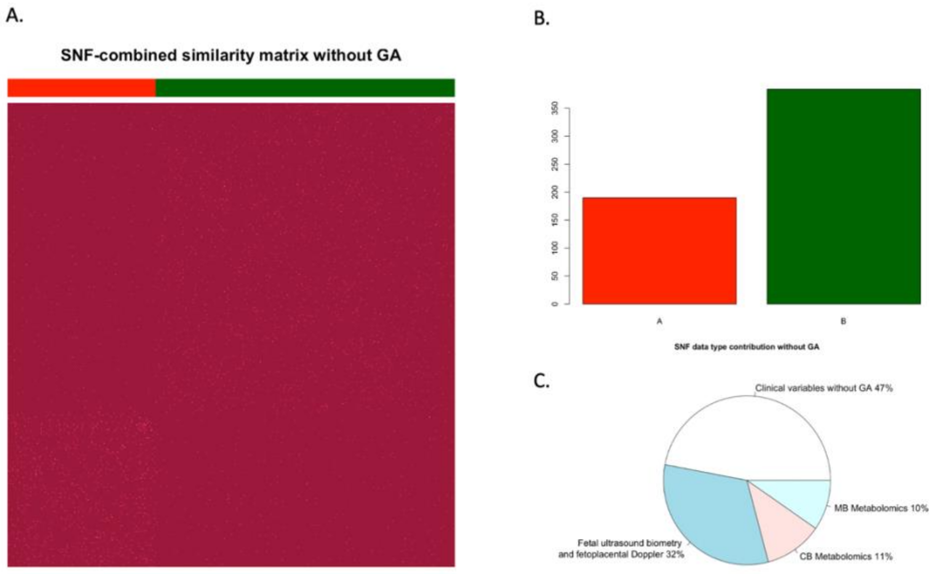

Figure S2: To test the influence of gestational age, a parallel similarity network fusion analysis was performed without including this variable in the clinical features. (A). Integrated networks were combined to generate a single fused network by implementing SNF to reveal patient clusters in the entire cohort, incorporating clinical and biological data. (B) The results demonstrated the existence of the two SNF clusters independently of including gestational age at recruitment. (C). SNF data contribution. When analyzing the contribution of each component, 47% of all patient similarities (edges) were due to two data clinical features, 32% due to maternal-fetal ultrasound parameters, and the remaining SNF edges were supported by maternal and cord blood metabolomics (21%), Related to Figure 3.

Figure S3. Log Cumulative Hazard plots examining the proportionality assumption of hazard to evaluate the covariate of Hazard proportionality.

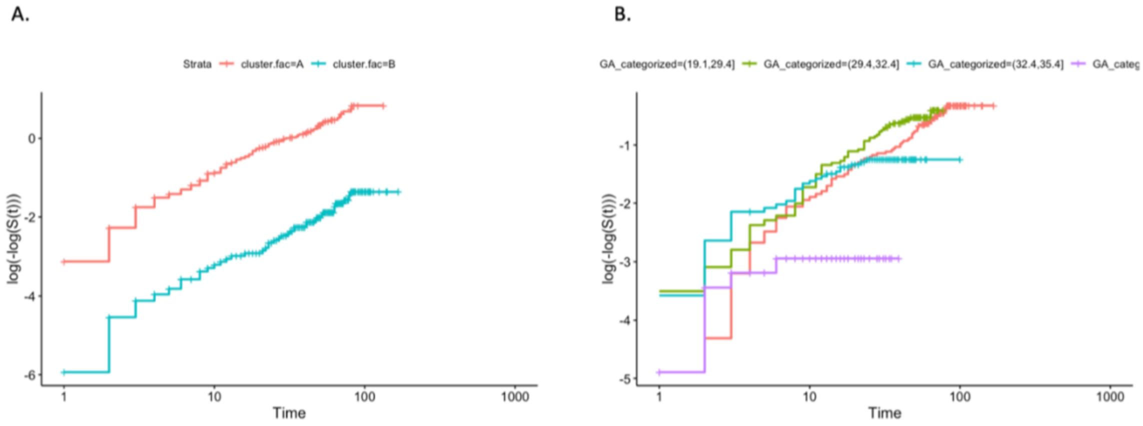

Figure S3: Log-log curve showing proportional hazard assumption according to each SNF cluster. Survival time over time is plotted in cluster A versus cluster B. The p values were not significant, demonstrating the assumption of proportionality, Related to Figure 5.

Table S1. Variables included in each of the domains used to generate the SNF clusters.

Clinical variables	Cord blood 1H-NMR metabolomics	Maternal blood 1H-NMR metabolomics
Gestational age at diagnosis (weeks)	Cholesterol VLDL	Cholesterol VLDL
Systolic BP at diagnosis (mmHg)	Cholesterol IDL	Cholesterol IDL
Diastolic BP at diagnosis (mmHg)	Cholesterol LDL	Cholesterol LDL
Mean BP at diagnosis (mmHg)	Cholesterol HDL	Cholesterol HDL
Maternal PlGF serum concentrations (MoM)	Triglycerides VLDL	Triglycerides VLDL
Maternal sFlt-1 serum concentrations (MoM)	Triglycerides IDL	Triglycerides IDL
Maternal sFlt-1/PlGF ratio (MoM)	Triglycerides LDL	Triglycerides LDL
	Triglycerides HDL	Triglycerides HDL
	VLDL Particle numbers (nmol/L)	VLDL Particle numbers (nmol/L)
	VLDL *Large* Particle numbers (nmol/L)	VLDL *Large* Particle numbers (nmol/L)
	VLDL *Medium* Particle numbers (nmol/L)	VLDL *Medium* Particle numbers (nmol/L)
	VLDL *Small* Particle numbers (nmol/L)	VLDL *Small* Particle numbers (nmol/L)
	Size of VLDL particles	Size of VLDL particles
	LDL Particle numbers (nmol/L)	LDL Particle numbers (nmol/L)
	LDL *Large* Particle numbers (nmol/L)	LDL *Large* Particle numbers (nmol/L)
	LDL *Medium* Particle numbers (nmol/L)	LDL *Medium* Particle numbers (nmol/L)
	LDL *Small* Particle numbers (nmol/L)	LDL *Small* Particle numbers (nmol/L)
	Size of LDL particles	Size of LDL particles
	HDL Particle numbers (nmol/L)	HDL Particle numbers (nmol/L)
**Maternal and fetal Ultrasound**	HDL *Large* Particle numbers (nmol/L)	HDL *Large* Particle numbers (nmol/L)
Estimated fetal weight (grams)	HDL *Medium* Particle numbers (nmol/L)	HDL *Medium* Particle numbers (nmol/L)
Estimated fetal weight (centile)	HDL *Small* Particle numbers (nmol/L)	HDL *Small* Particle numbers (nmol/L)
EFW z-velocity	Size of HDL particles	Size of HDL particles
Uterine artery PI Doppler at diagnosis (Z-score)	Ratio Total particles/HDL Particle numbers	Ratio Total particles/HDL Particle numbers
Umbilical artery PI Doppler at diagnosis (Z-score)	Ratio LDL particles/HDL *p*article numbers	Ratio LDL particles/HDL *p*article numbers
Middle cerebral artery PI Doppler at diagnosis (Z-score)	Alanine	Alanine
Cerebroplacental ratio at diagnosis (Z-score)	Citric acid	Citric acid
Ductus venosus PI Doppler at diagnosis (Z-score)	Creatine	Creatine
	Creatinine	Creatinine

Umbilical artery PI Doppler before delivery (Z-score)<br>Cerebroplacental ratio before delivery (Z-score)	Formic acid<br>Glucose<br>Glutamic acid<br>Glutamine<br>Glycine<br>3-Methylhistidine<br>Isoleucine<br>Lactic acid<br>Leucine<br>Lysine<br>Mannose<br>Methionine<br>2-oxoisovaleric acid<br>Phenylalanine<br>Pyruvic acid<br>Threonine<br>Tyrosine<br>Valine	Formic acid<br>Glucose<br>Glutamic acid<br>Glutamine<br>Glycine<br>3-Methylhistidine<br>Isoleucine<br>Lactic acid<br>Leucine<br>Lysine<br>Mannose<br>Methionine<br>2-oxoisovaleric acid<br>Phenylalanine<br>Pyruvic acid<br>Threonine<br>Tyrosine<br>Valine

Prespecified features selected as input variables for the SNF analysis, *Related to STAR methods.*

Abbreviations: BP= Blood pressure; HDL= High-density lipoproteins; IDL= Intermediate-density lipoproteins; LDL: Low-density lipoproteins; PI=Pulsatility index; PlGF= Placental growth factor; sFlt-1= soluble fms-like tyrosine kinase 1; VLDL: Very low-density lipoproteins

Table S2. Multinomial logistic regression classifier results.

Independent variables	Relative Risk ratio	95% CI
Gestational age at diagnosis (weeks)	0.74	(0.52 – 1.0)
Estimated fetal weight (grams)	0.31	(0.03 – 2.75)
Umbilical artery PI Doppler before delivery (Z-score)	5.12	(1.22 – 36.5)
Systolic BP at diagnosis (mmHg)	0.95	(0.89 – 1.0)
Uterine artery PI Doppler at diagnosis (Z-score)	1.07	(0.86 – 1.24)
Cerebroplacental ratio at diagnosis (Z-score)	0.35	(0.11 – 0.93)
Cerebroplacental ratio before delivery (Z-score)	1.96	(0.63 – 6.25)
Diastolic BP at diagnosis (mmHg)	1.14	(1.04 – 1.27)
Cord blood Creatine	0.94	(0.95 – 1.03)
Cord blood Glutamine	1.27	(1.14 – 1.48)
Cord blood Ratio LDL particles/HDL particle numbers	1.15	(1.06 – 1.27)

A classifier using the top 20 normalized mutual information (NMI) scores derived from the SNF analysis in the training dataset was constructed using multinomial logistic regression to predict new SNF groups generated in the test set. Related to Figure 6.

Abbreviations: BP= Blood pressure; HDL= High-density lipoproteins; LDL: Low-density lipoproteins; PI=Pulsatility index.

Table S3. Receiver operating characteristic curve analyses for obstetric outcomes.

Outcome	SNF Clusters AUC (95% CI)	GA diagnosis AUC (95% CI)	p-value	GA diagnosis + EFW centile + UA + UtA + CPR Doppler AUC (95% CI)	p-value*
Preeclampsia	0.83 (0.79 – 0.87)	0.63 (0.58 – 0.74)	<0.001	0.72 (0.67 – 0.77)	<0.001
Stillbirth	0.89 (0.81 – 0.97)	0.93 (0.67 – 0.98)	0.37	0.91 (0.84 – 0.98)	0.05
APO	0.71 (0.66 – 0.76)	0.64 (0.59 – 0.69)	0.001	0.70 (0.65 – 0.75)	0.57
Abnormal cord blood BNP	0.76 (0.69 – 0.84)	0.61 (0.54 – 0.68)	<0.001	0.72 (0.64 – 0.79)	0.33

SNF cluster membership probability consistently improved the prediction of preeclampsia over the clinical classification and had a similar performance for perinatal outcomes based on the area under the receiver operating characteristic (ROC) curve (AUC). Related to Figure 7.

Abbreviations: APO= Adverse perinatal outcomes; BNP= B-type natriuretic peptide; EFW= Estimated fetal weight; SNF= Similarity network fusion; UA= Umbilical artery; UtA=Uterine artery.

* P values comparing AUC curves of the membership probability of SNF cluster vs. clinical classification
